# Interplay of lncRNA H19/miR‐675 and lncRNA NEAT1/miR‐204 in breast cancer

**DOI:** 10.1002/1878-0261.12472

**Published:** 2019-03-14

**Authors:** Volkmar Müller, Leticia Oliveira‐Ferrer, Bettina Steinbach, Klaus Pantel, Heidi Schwarzenbach

**Affiliations:** ^1^ Department of Gynecology University Medical Center Hamburg‐Eppendorf Germany; ^2^ Department of Tumor Biology University Medical Center Hamburg‐Eppendorf Germany

**Keywords:** apoptosis, breast cancer, cell proliferation, lncRNAs, miRNAs, plasma

## Abstract

Long noncoding RNAs (lncRNAs) are frequently precursor RNAs of microRNAs (miRNAs) or act as competing endogenous RNAs (ceRNAs) to interact with miRNAs. To better understand the shared impact of lncRNAs and miRNAs in the regulatory post‐transcriptional network, we focused here on the relationships between (a) lncRNA H19 and miR‐675, (b) NEAT1 and miR‐204, and (c) HOTAIR and miR‐331 in plasma of early breast cancer (BC) patients. We quantified each RNA in plasma samples of 63 BC patients and 10 healthy women by quantitative real‐time PCR. In cell culture experiments, the influence of these noncoding RNAs (ncRNAs) on proliferation and apoptosis of BC cell line MCF‐7 was examined. Plasma levels of H19 (*P* = 0.030), NEAT1 (*P* = 0.030), and miR‐331 (*P* = 0.012) were deregulated in BC patients compared with healthy women. In both cohorts, the concentrations of H19 correlated with those of miR‐675 (*P* = 0.0001). Higher H19 (*P* = 0.001) along with lower miR‐675 (*P* = 0.007) levels and higher miR‐204 (*P* = 0.017) along with lower NEAT1 (*P* = 0.030) levels were detected in plasma of HER2‐positive patients compared with the other BC subgroups. Whereas the expression of HOTAIR was below the detection level, miR‐331 levels correlated with nodal status (*P* = 0.002) and recurrence (*P* = 0.012). In cell culture experiments, a competitive impact on cell proliferation and apoptosis by these ncRNAs was also documented. Our findings describe a relationship of the plasma levels of H19/miR‐675 and NEAT1/miR‐204 in the different BC subtypes; in addition, they reveal an interplay between these lncRNAs and miRNAs in the regulatory network in MCF‐7 cells, which should also be considered in the search for new diagnostic and therapeutic markers.

**Abbreviations**
BC: breast cancer
ceRNAs: competing endogenous RNAs
FFPE: formalin‐fixed paraffin‐embedded
HCC: hepatocellular carcinoma
HER2: human epidermal growth factor receptor 2
lncRNAs: long noncoding RNAs
miRNAs: microRNAs
ncRNAs: noncoding RNAs
NEAT1: nuclear paraspeckle assembly transcript 1
nt: nucleotides
TNBC: triple‐negative breast cancer

## 1 Introduction

At least 90% of the genome is transcribed into noncoding RNAs (ncRNAs) (Monroig *et al*., 2015; Qi *et al*., 2016). Based on their lengths, ncRNAs are classified into two main groups: Small ncRNAs, for example, microRNAs (miRNAs), comprise transcripts of < 200 nucleotides (nt) in length, while long ncRNAs (lncRNAs) include transcripts of 200–100 000 nt in size. These ncRNA molecules are shed into the bloodstream by various cell physiological events, such as apoptosis, necrosis, and active secretion. Apoptotic and necrotic cells release ncRNAs that form complexes with specific RNA‐binding proteins, such as AGO2 and HDL proteins, and/or integrate them in apoptotic bodies (Li *et al*., 2012; Schwarzenbach *et al*., 2014). ncRNAs are also released by exosomes – small membrane vesicles in size of approximately 100 nm that are actively secreted from multiple cell types, including dendritic cells, lymphocytes, and tumor cells, by exocytosis (Schwarzenbach, 2015, 2016b). This packaging protects ncRNAs against RNase digestion in the blood circulation and, consequently, provokes their highly stable circulation in blood (Mitchell *et al*., 2008). The levels of ncRNA can fluctuate in blood during the development and progression of cancer and display cancer stage‐specific expression profiles. In this regard, the quantification of aberrantly expressed ncRNAs in blood as well as in other liquid biopsies (e.g., urine, saliva, pleural effusion, and cerebrospinal fluid) could serve as an assay for the development of new blood‐based biomarkers for cancer detection and course of the disease. In addition, their screening and subsequent functional analyses may deliver information on their target molecules and deregulated signaling pathways which they are involved in. Therefore, understanding of their mechanism of action could improve treatment regimens and establish new targeted therapies (Schwarzenbach, 2014).

MicroRNAs are single‐stranded and about 25‐nt‐long ncRNA molecules. Their binding to two proteins (GW182 protein and AGO2) generates a complex called miRNA‐induced silencing complex (miRISC) (Fabian and Sonenberg, 2012; Höck and Meister, 2008). In this complex, miRNAs bind to complementary sequences in the 3′ UTR of their target mRNAs and repress post‐transcriptionally their translation into protein (Bartel, 2009). In almost the same manner, lncRNAs suppress the translation of specific mRNAs, especially adjacent protein‐encoding genes. LncRNAs can also modify gene expression through recruitment of histone modification complexes. Moreover, they are involved in alternative splicing and nuclear import (Schmitt and Chang, 2016; Schwarzenbach, 2016b). A particular feature is their interaction with miRNAs (Liu *et al*., 2014; Lu *et al*., 2016; Yoon *et al*., 2014) and/or to serve as precursors of miRNAs (Schwarzenbach, 2016a). Thus, lncRNAs can act as ceRNAs (competing endogenous RNAs) to de‐repress gene expression by competing with miRNAs for interaction with shared target mRNAs, or they produce miRNAs, leading to repression of their target mRNAs. This miRNA/lncRNA crosstalk modulates gene expression patterns that drive physiological and pathological processes (Yoon *et al*., 2014), implicating that ncRNAs could be of significant relevance in breast cancer (BC).

Of particular interest for BC might be the detection of H19 which was the first lncRNA identified as a riboregulator (Brannan *et al*., 1990). H19 is involved in embryonic development, paternally imprinted and accordingly maternally expressed. Modifications of imprinting cause its aberrant expression and are responsible for the development of diseases. To date, numerous studies have reported its key regulatory functions in tumor development and progression (Barsyte‐Lovejoy *et al*., 2006; Raveh *et al*., 2015; Vennin *et al*., 2015; Zhang *et al*., 2016). In 2007, Cai and Cullen demonstrated that this oncofetal lncRNA is the precursor RNA of miR‐675 which is transcribed from the first exon of H19 (Cai and Cullen, 2007; Tsang *et al*., 2010). The association of H19 with tumorigenesis and invasion is assumed to be owed to the regulation of its integrated carcinogenic miR‐675 (Schwarzenbach, 2016a; Vennin *et al*., 2015). Also in 2007, HOX antisense intergenic RNA (HOTAIR) was identified. Rinn *et al*. found that this lncRNA is at the boundary of two diametrical chromatin domains in the HOXC locus and transcribed in an antisense manner. It plays a critical role in chromatin dynamics and regulates gene silencing by its association with histone methylation (Rinn *et al*., 2007). Moreover, HOTAIR interacts with miRNAs to link miRNAs and the post‐transcriptional network in cancer pathogenesis. In gastric cancer, HOTAIR forms complementary base pairing with miR‐331 and inversely correlates with the occurrence of miR‐331. Acting as a ceRNA of miR‐331, it regulates the expression of human epithelial growth factor receptor 2 (HER2) through competition for miR‐331 (Liu *et al*., 2014). A physical interaction was also reported between nuclear paraspeckle assembly transcript 1 (NEAT1) and miR‐204, forming a reciprocal repression feedback loop in nasopharyngeal carcinoma (Lu *et al*., 2016). NEAT1 is localized specifically to nuclear bodies, called paraspeckles, which are irregularly shaped compartments in the interchromatin of the nucleus and involved in the development of the mammary gland. Loss of NEAT1 leads to the disintegration of paraspeckles (Standaert *et al*., 2014).

In the present study, we analyzed the associations of lncRNAs H19, NEAT1, and HOTAIR with miR‐675, miR‐204, and miR‐331, respectively, in the plasma of BC patients and the BC cell line MCF‐7. These investigations shall contribute to a better understanding on the shared impact of these lncRNA/miRNA pairs on regulation of the expression of their target mRNAs whose protein products are involved in BC‐associated signaling pathways.

## 2 Materials and methods

### 2.1 Plasma samples

Blood samples from 63 early BC patients with no signs of distant metastases were collected before surgery from June 2013 to August 2016 at the Department of Gynecology of the University Medical Center Hamburg‐Eppendorf. All patients gave written informed consent to examine their blood and review their medical records according to our investigational review board and ethics committee guidelines. Blood collection and experiments were performed in compliance with the Helsinki Declaration and were approved by the ethics committee (Ethik‐Kommission der Ärztekammer, Hamburg, Germany, PV5392). Regarding blood processing, uniform management concerning the specific described protocols was performed. In Table 1, patient characteristics at the time of BC diagnosis are summarized. Additionally, blood samples were collected from 10 healthy women and stored under the same conditions.

**Table 1:** Patient characteristics at the time of diagnosis of BC

Parameters	Patients (%)
Patients	63 (100)
Mean/Median Age	56/55 years (range: 30–91 years)
Tumor stage	
cT1	36 (57)
cT2–3	27 (43)
Grading	
G1, G2	30 (49)
G3	31 (51)
Lymph node metastasis	
N0	49 (78)
N1	14 (22)
Lymph invasion	
No	55 (87)
Yes	8 (13)
ER status	
Positive	38 (60)
Negative	25 (40)
PR status	
Positive	33 (52)
Negative	30 (48)
Triple‐negative	
No	46 (73)
Yes	17 (27)
HER2‐positive	
No	55 (87)
Yes	8 (13)
Recurrence	
No	56 (93)
Yes	4 (7)

For the preparation of plasma, blood samples were centrifuged at 2700 ***g*** for 10 min. Then, the supernatant was centrifuged at 13 500 ***g*** for 10 min, to remove residual cells.

### 2.2 Extraction of RNA

RNA was extracted from plasma using the miRNeasy kit (Qiagen, Hilden, Germany) corresponding to the manufacturer′s protocol. Briefly, 1 mL QIAzol Lysis Reagent was mixed with 200 μL plasma. Following incubation at RT for 5 min, 3.5 μL of 100 fmol cel‐miR‐39 was added as an exogenous spike‐in control to the lysate. RNA precipitation was carried out with 200 μL chloroform and 900 μL 100% ethanol in two separate steps. Then, 700 μL of the samples was added to an RNeasy MinElute spin column and centrifuged at 9300 ***g*** at RT for 15 s. Following washing the columns with 700 μL RWT and 500 μL RPE buffer, and centrifugation at 9300 ***g*** at RT for 15 s, RNA precipitation was carried out with 500 μL of 80% ethanol. RNA was eluted from the column with 14 μL RNase‐free water.

### 2.3 cDNA synthesis and preamplification of lncRNAs

Extracted RNA was reverse transcribed into cDNA and preamplified using the RT2 PreAMP cDNA Synthesis Kit (Qiagen). To eliminate genomic DNA, 8 μL RNA was incubated with 2 μL GE buffer at 42 °C for 5 min. Reverse transcription was carried out with this 10 μL mix supplemented with 4 μL BC3 buffer, 1 μL control P2, 1 μL cDNA Synthesis Enzyme Mix, 1 μL RNase, and 3 μL RNase‐free water, incubated at 42 °C for 30 min, and stopped at 95 °C for 5 min on a MJ Research PTC‐200 Peltier Thermal Cycler (Global Medical Instrumentation, Ramsey, MN, USA). For preamplification, 5 μL cDNA was mixed with 12.5 μL RT2 PreAMP PCR Mastermix and 7.5 μL RT2 PreAMP Pathway Primer Mix specific for H19, HOTAIR, and NEAT1. Cycling conditions comprised 95 °C for 10 min to activate HotStart DNA Taq polymerase and 15 cycles of 95 °C for 15 s and 60 °C for 2 min on a MJ Research PTC‐200 Peltier Thermal Cycler (Global Medical Instrumentation).

### 2.4 Quantitative real‐time PCR of lncRNAs

For this experiment, lncRNA‐specific RT^2^qPCR Primer Assays (Qiagen) for β‐actin (reference RNA), H19, NEAT1, and HOTAIR were used. In a 20 μL reaction, 1 μL preamplified product was mixed with 10 μL RT^2^ SYBR Green Mastermix and 0.8 μL lncRNA‐specific RT^2^ lncRNA qPCR assay on a twin‐tec real‐time PCR plate (Eppendorf, Hamburg, Germany). The quantitative real‐time PCR was carried out at 95 °C for 10 min and in 40 cycles at 95 °C for 15 s and 60 °C for 60 s on an Applied Biosystems 7500 fast real‐time PCR device (Applied Biosystems, Darmstadt, Germany).

### 2.5 cDNA synthesis and preamplification of miRNAs

Extracted RNA was reverse transcribed into cDNA and preamplified using the Creating Custom RT and Preamplification Pool and TaqMan MicroRNA assays (Thermo Fisher Scientific, Darmstadt, Germany). Three microlitre RNA was reverse transcribed into cDNA with 6 μL RT primer pool, 0.30 μL 100 mm dNTPs, 3 μL 50 U·μL^−1^ MultiScribe Reverse Transcriptase, 1.5 μL RT buffer, and 0.19 μL 20 U·μL^−1^ RNase inhibitor. The 15 μL reaction was incubated on ice for 5 min and then at 16 °C for 30 min, at 42 °C for 30 min, and at 85 °C for 5 min on a MJ Research PTC‐200 Peltier Thermal Cycler (Global Medical Instrumentation).

One microlitre cDNA was preamplified in a 10 μL reaction containing 5 μL TaqMan PreAmp Master Mix and 1.5 μL Custom PreAmp primer pool (Thermo Fisher Scientific). PCR was run on a MJ Research PTC‐200 Peltier Thermal Cycler (Global Medical Instrumentation): one cycle at 95 °C for 10 min, 55 °C for 2 min, 72 °C for 2 min; 16 cycles at 95 °C for 15 s, 60 °C for 4 min; and a terminal cycle at 99.9 °C for 10 min. To avoid false‐positive data (e.g., primer dimer formation or unspecific PCR products), a negative control without any templates was included from the starting point of reverse transcription.

### 2.6 Quantitative TaqMan real‐time PCR of miRNAs and data normalization

For quantitative real‐time PCR, the miRNA‐specific TaqMan miRNA assays (Thermo Fisher Scientific) for miR‐484 (reference miRNA), miR‐675, miR‐204, and miR‐331 were used (Table S1). In a 10 μL reaction, 1.5 μL preamplified product was mixed with 10 μL TaqMan Universal PCR Master Mix No AmpErase UNG and 1 μL miRNA‐specific TaqMan MicroRNA Assay Mix on a twin‐tec real‐time PCR plate (Eppendorf). The quantitative real‐time PCR was performed at 95 °C for 10 min and in 40 cycles at 95 °C for 15 s and 60 °C for 60 s on an Applied Biosystems 7500 fast real‐time PCR device (Applied Biosystems).

As there is no consensus concerning the data normalization, we chose miR‐484 and β‐actin as references to normalize our miRNA and lncRNA data, respectively, because these RNAs showed the smallest variations. The obtained data of miRNA and lncRNA expression levels were calculated and evaluated by the Δ*C*_t_ method as follows: Δ*C*_t_ = mean value *C*_t_ (reference miR‐484 + cel‐miR‐39) − mean value *C*_t_ (miRNA of interest) and Δ*C*_t_ = mean value *C*_t_ (reference β‐actin) − mean value *C*_t_ (lncRNA of interest), respectively. The relative miRNA and lncRNA levels refer to the value of $2^{\Delta C_{t}}$ (Table S2) (Schwarzenbach *et al*., 2015).

### 2.7 Cell culture and transient transfection

MCF‐7 cells were purchased from ATCC (American Type Culture Collection, Wesel, Germany) and authenticated by the Leibniz Institute DSMZ (Deutsche Sammlung von Mikroorganismen und Zellkulturen GmbH, Braunschweig, Germany). The cells were cultured in RPMI 1640, supplemented with 10% FBS (PAA, Laboratories, Cölbe, Germany) under standard conditions (37 °C, 5% CO_2_, humidified atmosphere), and regularly tested for mycoplasma contamination (Minerva Biolabs, Berlin, Germany). They were seeded into 96‐well plates at a density of 5000 cells per well in triplicate and transfected with siRNAs H19.1, H19.2, H19.3, H19.4 (FlexiTube GeneSolution for H19), siRNAs NEAT1.1, NEAT1.2, NEAT1.3, NEAT1.4 (FlexiTube GeneSolution for NEAT1), double‐stranded miScript miRNA mimic of hsa‐miR‐204 and miR‐675, single‐stranded miScript inhibitor of miR‐675, miScript inhibitor negative control, or AllStars negative control small interfering RNA (negative control) at final concentrations of 10 and 20 nm, together with 0.75 μL HiPerFect^®^ Transfection Reagent (Qiagen). The sequences of siRNAs and miScripts are shown in Table S1.

### 2.8 MTT assay

Following 24, 48, and 72 h of transfection with miRNA mimics, inhibitors, or negative controls, cells were incubated with 20 μL 5 mg·mL^−1^ MTT (thiazolyl blue tetrazolium bromide; Sigma‐Aldrich, Saint Louis, MO, USA) in PBS at 37 °C for 3 h. Then, the cells were lysed with lysis buffer (4 mm HCl, 0.1% NP40 in isopropanol), to solubilize the colored crystals. OD (optical density) was measured at 540 and 650 nm (reference) on a microplate reader (Tecan, Männerdorf, Switzerland). Each experiment contained three replicate wells and was repeated three times.

### 2.9 Apoptosis assay and flow cytometry

To examine the effect of the ncRNAs on apoptosis, MCF‐7 cells were additionally treated with the topoisomerase I inhibitor camptothecin (Biovision, Milpitas, CA, USA). Twenty‐four hours after transfection, MCF‐7 cells on a six‐well plate were treated with 4.8 μm camptothecin for 4 h and incubated for further 24 h to induce apoptosis. Nontransfected and transfected cells untreated or treated with camptothecin were incubated with 3 μL Annexin‐V‐FITC (BD Biosciences, San Jose, CA, USA) and 5 μL propidium iodide (Sigma‐Aldrich) in the dark at 4 °C for 15 min. Following incubation, 400 μL Annexin‐FITC binding buffer (0.1 m Hepes pH 7.4, 1.4 m NaCl, 25 mm CaCl_2_ in PBS) was added to the cells. The cells were then analyzed on a FACSCanto II flow cytometer (BD Biosciences).

### 2.10 Statistical analyses

Statistical analyses were performed using spss software package, version 22.0 (SPSS Inc., Chicago, IL, USA). Because of the skewed distribution of miRNA and lncRNA concentrations, differences in group levels for nonparametric comparisons were bivariately assessed by univariate analyses of the Mann–Whitney *U*‐test of two independent variables. Bivariate analyses of the Spearman‐Rho test were also used. Changes in proliferation and apoptosis were calculated by using ANOVA Tukey's HSD test. Missing data were handled by pairwise deletion. A *P*‐value < 0.05 was considered as statistically significant. All *P*‐values are two‐sided. Due to the explorative nature of the study, no formal adjustment for multiple testing was performed.

## 3 Results

### 3.1 Quantification of lncRNAs and miRNAs in blood plasma

The aim of our study was to investigate whether there is a relationship of the expression levels of miRNAs with those of their precursor or interaction partner miRNAs in plasma of BC patients. For quantification of the lncRNA/miRNA pairs, we selected H19/miR‐675, NEAT1/miR‐204, and HOTAIR/miR‐331, and applied the most commonly used kits from Qiagen and Thermo Fisher Scientific. Although we have long‐standing experience on liquid biopsy analyses, our attempts of establishing and comparing these kits showed that both kits had different specificities. The miRNA‐specific TaqMan miRNA assay from Thermo Fisher Scientific provided most solid data on plasma miRNAs, but not on plasma lncRNAs. Inversely, the Qiagen kit delivered most solid data on plasma lncRNAs, but not on plasma miRNAs. Therefore, to obtain reliable data and avoid false‐positive data (e.g., primer dimer products), we were compelled to quantify plasma miRNAs with the Thermo Fisher Scientific and lncRNAs with the Qiagen kit. However, since the measurements provide relative data, the values of lncRNAs and miRNAs can be evaluated together. Unfortunately, our measurements showed that the expression levels of HOTAIR were too low in most of our plasma samples, to be correctly interpreted. Since only a few plasma samples delivered detectable values of plasma HOTAIR, we excluded these analyses from our study. On the other hand, the quantification of miR‐675 with its precursor lncRNA H19, miR‐204 with its ceRNA NEAT1, and miR‐331 provided robust data in all plasma samples (Table S2).

### 3.2 Deregulated plasma levels of H19, NEAT1, and miR‐331 in BC patients

**Figure 1:**
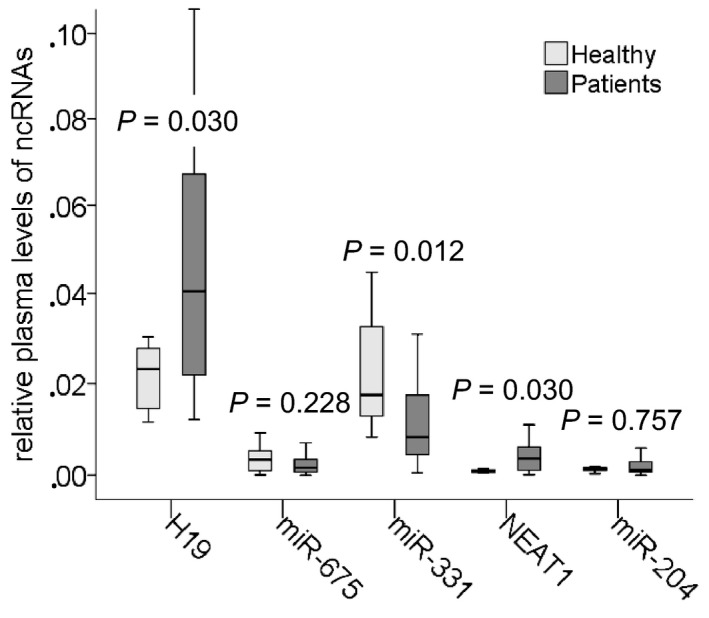
Deregulated plasma levels of H19, miR‐331, and NEAT1 in BC patients. The box plot compares the levels of H19, miR‐675, miR‐331, NEAT1, and miR‐204 in plasma of 63 BC patients with those in 10 healthy women.

At first, we quantified and compared the relative plasma levels of H19, miR‐675, NEAT1, miR‐204, and miR‐331 in 63 BC patients with those in 10 healthy women. The plasma levels of H19 (*P* = 0.030) and NEAT1 (*P* = 0.030) were upregulated, and those of miR‐331 (*P* = 0.012) were downregulated in BC patients compared with healthy women. In contrast, the levels of miR‐675 (*P* = 0.228) and miR‐204 (*P* = 0.757) were similar in both cohorts (Fig. 1). When we compared the ncRNA levels in the single patient subgroups with those in healthy women, we found that the plasma levels of H19 were higher in patients with lymph node‐negative status (*P* = 0.033) and HER2‐positive patients (*P* = 0.008) than in healthy women, indicating that the aberrant expression of H19 is rather associated with early and HER2‐positive tumors. Compared with healthy women, the plasma levels of NEAT1 were higher in lymph node‐negative patients (borderline *P* = 0.045) as well as in lymph node‐positive patients (*P* = 0.034) and particularly, in triple‐negative (TNBC) patients (*P* = 0.002), indicating that the deregulation of NEAT1 was rather associated with more aggressive tumors. Finally, the plasma levels of miR‐331 were lower in patients with lymph node‐negative status (*P* = 0.031) as well as with lymph node metastases (*P* = 0.002) and HER2‐positive patients (*P* = 0.043) than in healthy women, indicating that the decrease in plasma miR‐331 was particularly associated with advanced tumors. As observed in the entire BC patient cohort, the expression levels of miR‐675 and miR‐204 were also not deregulated in the single patient subgroups (Table 2).

**Table 2:** Associations of plasma levels of circulating lncRNAs and miRNAs with clinicopathological parameters of BC patients at diagnosis

Parameters	Plasma lncRNAs and miRNAs – *P* values				
	H19	miR‐675	NEAT1	miR‐204	miR‐331
Correlations*					
lncRNA/miRNA	**0.007 (coefficient: 0.334)**		0.070 (coefficient: −0.293)		–
BC patient subgroups vs. healthy women**					
All	**0.030** a	0.228	**0.030** a	0.757	**0.012** b
Neg. nodal status	**0.033** a	0.067	**0.045** a	0.632	**0.031** b
Pos. nodal status	0.083	0.403	**0.034** a	0.902	**0.002** b
HER2‐pos. (ER‐neg./PR‐neg.)	**0.008** a	0.375	0.412	0.051	**0.043** b
TNBC (ER‐neg./PR‐neg./HER2‐neg.)	0.660	0.060	**0.002** a	0.820	0.264
Differences between the BC patient subgroups**					
Pos. vs. neg. lymph node status	0.430	**0.004** a	0.092	0.684	0.192
TNBC vs. the other subgroups in which one or two or all receptors (ER, PR, HER2) are pos.	**0.012** a	0.067	0.122	0.795	0.129
HER2‐pos. (ER‐neg./PR‐neg.) vs. the other receptor statuses	**0.001** a	**0.007** b	**0.030** b	**0.017** a	0.628
ER‐neg. vs. ER‐pos.	0.075	**0.048**	0.729	0.253	**0.034**
PR‐neg. vs. PR‐pos.	0.148	0.558	0.576	0.649	**0.022**
ER‐neg./PR‐neg. vs. the other subgroups	0.075	**0.044**	0.877	0.253	0.072
Recurrence^c^ vs. primary	0.806	0.356	0.341	0.631	**0.012** a

^a^Upregulated, ^b^Downregulated, ^c^At diagnosis. Significances in bold calculated by *Spearman‐Rho and **Mann–Whitney *U*‐test.

### 3.3 Differences in plasma levels of H19/miR‐675, NEAT1/miR‐204, and miR‐331 between the BC subgroups

The elevated plasma levels of H19 did not only differ HER2‐positive patients from healthy women (*P* = 0.008) but also differ HER2‐positive patients from the other patient subgroups (*P* = 0.001), indicating an association of plasma H19 with tumors that overexpress HER2. Although plasma miR‐675 was not deregulated in the entire BC group as well as in the single BC subgroups, its levels differed between lymph node‐negative and lymph node‐positive BC patients (*P* = 0.004) as well as HER2‐positive BC and the other subgroups (*P* = 0.007). Strikingly, H19 and miR‐675 displayed opposite levels: Those of H19 were increased (*P* = 0.001) and those of miR‐675 were decreased (*P* = 0.007) in HER‐positive compared with the other subgroups. Alike, NEAT1 and miR‐204 also showed opposite levels in HER2‐positive patients: Those of NEAT1 were decreased (*P* = 0.030), and those of miR‐204 were increased (*P* = 0.017). Interestingly, the plasma levels of miR‐331 were associated with ER (*P* = 0.034) and PR (*P* = 0.022) status. Our BC patient cohort contained a small subgroup of recurrent patients at diagnosis who got a relapse in average of 16 years (range from 12 to 22 years). In this subgroup, the plasma levels of miR‐331 were upregulated (*P* = 0.012, Table 2).

### 3.4 Significant association of plasma levels of H19 with miR‐675

**Figure 2:**
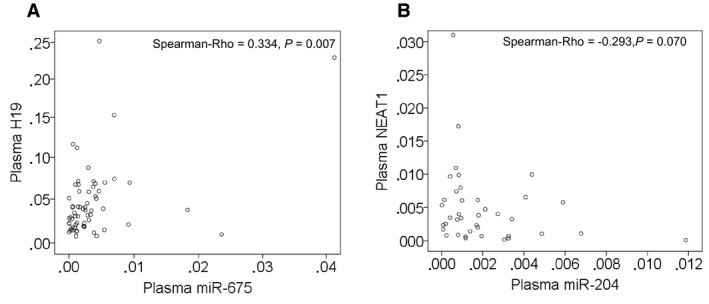
Significant relationship of the plasma levels of H19 with those of miR‐675. The scatter plots show the correlations of the levels of H19 with those of miR‐675 (A) and the levels of NEAT1 with those of miR‐204 (B) in plasma of BC patients, as determined by the Spearman‐Rho test.

As shown by the scatter plot in Fig. 2 and summarized in Table 2, there was a significant, positive correlation between the plasma levels of H19 and miR‐675 (*P* = 0.007) in both cohorts of BC patients and healthy women, supporting the fact that miR‐675 is a derivative of H19 (Fig. 2A). No correlation between the plasma levels of NEAT1 and miR‐204 (*P* = 0.070) could be observed, indicating that the link of NEAT1 as ceRNA to miR‐204 does not explicitly mean a reciprocal dependency of their concentrations (Fig. 2B).

### 3.5 Impact of H19/miR‐675 and NEAT1/miR‐204 on cell proliferation and apoptosis

To analyze the functions of lncRNAs (H19 and NEAT1) those relatively basal levels are high in MCF‐7 cells, they were inhibited in this cell line by the corresponding siRNAs. We performed our analysis with four siRNAs of H19 (H19.1, H19.2, H19.3, and H19.4 inhibitors) that bind to different sequences of H19 and four siRNAs of NEAT1 (NEAT1.1, NEAT1.2, NEAT1.3, and NEAT1.4 inhibitors) that bind to different sequences of NEAT1. The use of siRNAs of NEAT1.1, NEAT1.2, H19.1, and H19.4 showed the best results, and their impacts on cell proliferation and apoptosis are depicted. We also transfected both miR‐675 mimic and miR‐675 inhibitor into MCF‐7 cells to analyze their influence on the interplay with its derivative of H19, permitting us to better determine the single effects of H19 and miR‐675. In addition, we overexpressed miR‐204 by miR‐204 mimic because its relatively basal levels were about 20 times lower than those of the other ncRNAs and because of the reciprocal repression of NEAT1 and miR‐204. This allows us to intensify and localize the effect of miR‐204 that could otherwise be influenced by the inhibitory effect of NEAT1.

**Figure 3:**
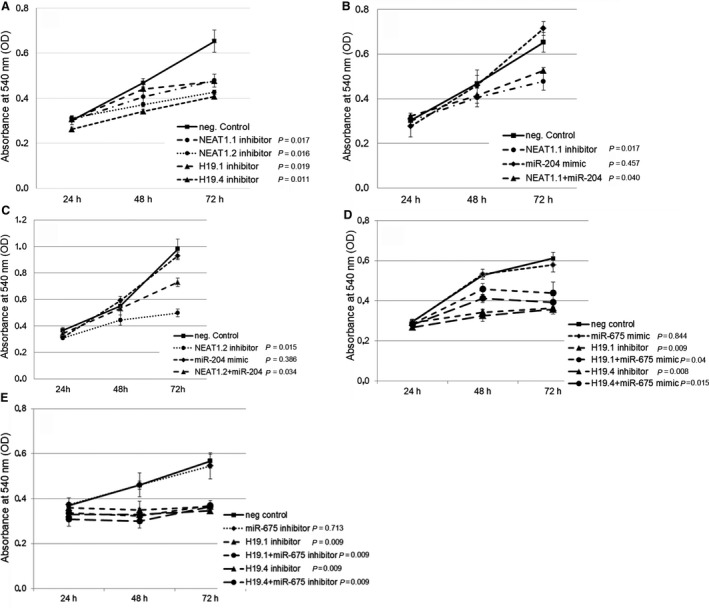
Effects on cell proliferation by H19/miR‐675 and NEAT1/miR‐204. MCF‐7 cells were transiently transfected with a miScript inhibitor negative control, inhibitors of NEAT1.1 or NEAT1.2, and inhibitors of H19.1 or H19.4 (A), with miScript inhibitor negative control, AllStars negative control small interfering RNA, NEAT1.1 inhibitor, miR‐204 mimic and both, NEAT1.1 inhibitor plus miR‐204 mimic (B), with miScript inhibitor negative control, AllStars negative control small interfering RNA, NEAT1.2 inhibitor, miR‐204 mimic and both, NEAT1.2 inhibitor plus miR‐204 mimic (C), with miScript inhibitor negative control, AllStars negative control small interfering RNA, inhibitors of H19.1 or H19.4, or miR‐675 mimic, H19.1 or H19.4 inhibitor plus miR‐675 mimic (D), with miScript inhibitor negative control, AllStars negative control small interfering RNA, inhibitors of H19.1 or H19.4, or miR‐675 inhibitor, H19.1 or H19.4 inhibitor plus miR‐675 inhibitor (E). After 24, 48, and 72 h, cells were treated with MTT. Cell proliferation was measured at an absorbance of 540 nm. The experiments were repeated two to three times and showed similar results, indicated by error bars representing standard deviations. The *P*‐values refer to the comparison of the indicated ncRNAs with the average of the two neg. controls which displayed similar values.

Hence, we transiently transfected MCF‐7 cells with siRNAs of H19.1, H19.4, NEAT1.1, and NEAT1.2, as well as miR‐675 mimic or inhibitor and miR‐204 mimic. MCF‐7 cells transfected with miR‐204 mimic harbored about 100 times higher levels of miR‐204. Inhibition of NEAT1.1 (*P* = 0.017), NEAT1.2 (*P* = 0.016), H19.1 (*P* = 0.019), and H19.4 (*P* = 0.011) had the strongest inhibitory effect on cell proliferation, suggesting that NEAT1 and H19 stimulated cell proliferation (Fig. 3A). MiR‐204 did not affect cell proliferation (*P* = 0.457). However, overexpression of miR‐204 could alleviate the inhibitory effect on cell proliferation by siRNA NEAT1.1 (from *P* = 0.017 to *P* = 0.040, Fig. 3B) and siRNA NEAT1.2 (from *P* = 0.015 to *P* = 0.034, Fig. 3C). Transfection of miR‐675 mimic had no effect on cell proliferation (*P* = 0.844). Alike the interplay between NEAT1 and miR‐204, overexpression of miR‐675 could alleviate the inhibitory effect on cell proliferation by siRNA H19.1 (from *P* = 0.009 to *P* = 0.04) and siRNA H19.4 (from *P* = 0.008 to *P* = 0.015, Fig. 3D). Inhibition of miR‐675 had also no effect on cell proliferation (*P* = 0.713) and could also not increase the inhibitory effect of siRNA H19.1 and siRNA H19.4 (*P* = 0.009, Fig. 3E).

**Figure 4:**
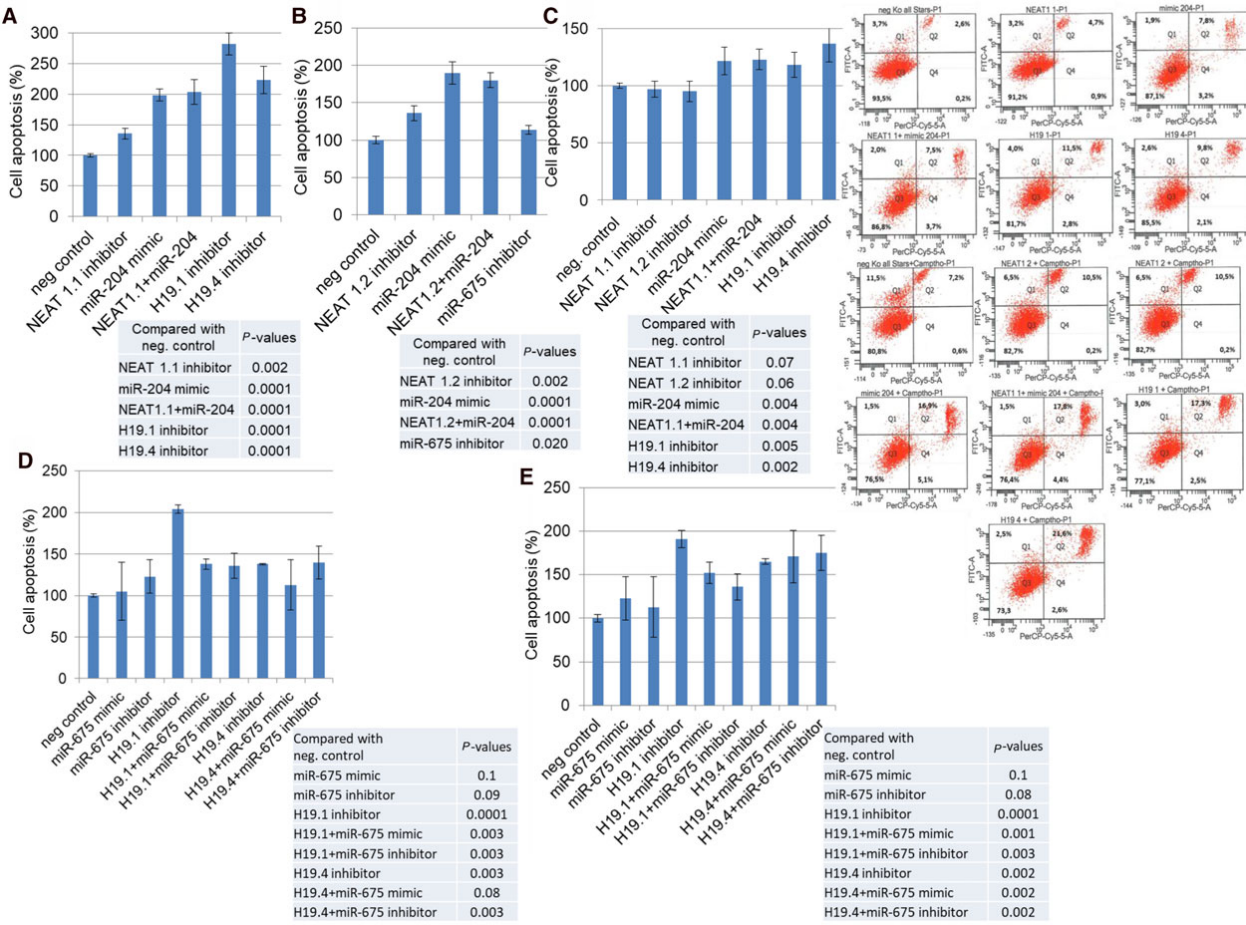
Effects on apoptosis by H19/miR‐675 and NEAT1/miR‐204. MCF cells were transfected with miScript inhibitor negative control, AllStars negative control small interfering RNA, NEAT1.1 inhibitor and/or miR‐204 mimic, and inhibitors of H19.1 and H19.4 (A), with miScript inhibitor negative control, AllStars negative control small interfering RNA, NEAT1.2 inhibitor and/or miR‐204 mimic, and miR‐675 (B), and additionally treated with the topoisomerase I inhibitor camptothecin (C), with miScript inhibitor negative control, AllStars negative control small interfering RNA, inhibitors of H19.1 or H19.4, miR‐675 mimic or inhibitor and both, H19.1 inhibitor plus miR‐675 mimic or inhibitor and H19.4 inhibitor plus miR‐675 mimic or inhibitor (D), and additionally treated with the topoisomerase I inhibitor camptothecin (E). The bar charts summarize the data on the apoptotic effect by these lncRNAs and miRNAs as derived from the FACSCanto II device. The experiments were repeated two times and showed similar results, indicated by error bars representing standard deviation. The *P*‐values refer to the comparison of the indicated ncRNAs with the average of the two neg. controls which displayed similar values. Below the bar charts, exemplary diagrams of the FACS analyses using MCF cells untreated and treated with Camptothecin are shown. Cells were labeled with Annexin‐V‐FITC and propidium iodide. Cell fragments only positive for propidium iodide can be found in the upper left corner (Q1). Late apoptotic as well as necrotic cells can be found in the upper right corner (Q2), since they are positive for Annexin and propidium iodide. Living cells are negative for Annexin and propidium iodide and, therefore, can be found in the lower left corner (Q3). Early apoptotic cells are only positive for Annexin and located in the lower right corner (Q4). The size for each population (%) is given in the corresponding area.

The effect of these ncRNAs was also examined on apoptosis in transfected MCF‐7 cells untreated and treated with camptothecin. Camptothecin is used in cancer chemotherapy to induce apoptosis. In untreated MCF‐7 cells (without Camptothecin), siRNA NEAT1.1 (*P* = 0.002), miR‐204 mimic (*P* = 0.0001), siRNA H19.1 (*P* = 0.0001), and siRNA H19.4 (*P* = 0.0001) induced cell apoptosis. Inhibition of NEAT1.1 could not further increase the strong stimulatory effect by miR‐204 mimic on apoptosis (*P* = 0.0001, Fig. 4A). Similar effects on cell apoptosis could be observed for the variant NEAT1.2 without (*P* = 0.002) and with cotransfected miR‐204 (*P* = 0.0001, Fig. 4B). The additional treatment of the transfected cells with camptothecin showed that the inhibition of NEAT1.1 (*P* = 0.07) and NEAT1.2 (*P* = 0.06) could not enhance the apoptotic effect of camptothecin. However, overexpression of miR‐204 (*P* = 0.004) and the inhibition of H19.1 (*P* = 0.005) and H19.4 (*P* = 0.002) could further increase the apoptosis induced by camptothecin (Fig. 4C). Besides, overexpression (*P* = 0.1) and inhibition (*P* = 0.09) of miR‐675 had also no impact on apoptosis in untreated MCF‐7 cells. However, overexpression of miR‐675 mimic decreased the apoptotic effect by siRNA H19.1 (from *P* = 0.0001 to *P* = 0.003) and H19.4 (from *P* = 0.003 to *P* = 0.08). Discrepant data were obtained for the effect of miR‐375 inhibitor on siRNA H19. Curiously, miR‐675 inhibitor also alleviated the apoptotic effect by siRNA H19.1 (from *P* = 0.0001 to *P* = 0.003), but did not affect siRNA H19.4 (*P* = 0.003, Fig. 4D). Similar data were obtained for miR‐675 in camptothecin‐treated MCF‐7 cells Fig. 4E).

These findings show that NEAT1 and H19 have oncogenic features. They increased cell proliferation and decreased apoptosis, while miR‐204 induced apoptosis. Surprisingly, miR‐675 had only an effect on both cell processes in MCF‐7 cells cotransfected with H19.

## 4 Discussion

In the current study, we demonstrated the significant, positive relationship of the plasma levels of miR‐675 with those of its precursor lncRNA H19 in BC patients. In particular, the plasma levels of H19 seem to be associated with early HER2‐positive BC, since they were significantly higher in lymph node‐negative and HER2‐positive patients than in healthy women. In contrast to its precursor H19, the levels of miR‐675 were not deregulated in the entire BC cohort and subgroups. In our experimental model, H19 stimulated cell proliferation and inhibited apoptosis, whereas miR‐675 did not affect both cell processes, but affected cotransfected H19. Our findings suggest that in our study, H19 displayed oncogenic features in contrast to miR‐675. As expected, there was no correlation of the plasma levels between NEAT1 and miR‐204, since their formation of a reciprocal repression feedback loop does not necessarily postulate such a relationship. High plasma levels of NEAT1 were most significantly associated with lymph node‐positive and TNBC patients, suggesting rather its role in advanced BC. Inhibition of NEAT1 inhibited cell proliferation and increased apoptosis, indicating that NEAT1 may stimulate cell proliferation and impede apoptosis. However, overexpression of miR‐204 could alleviate the stimulatory effect on cell proliferation by NEAT1. Our findings suggest that NEAT1 acts as an oncogene, whereas miR‐204 acts as a tumor suppressor. Finally, our data on plasma HOTAIR were not analyzable. As recognizable by real‐time amplification curves, TaqMan PCR frequently delivered false‐positive PCR products containing primer dimer products. So, the data on plasma miR‐331 were analyzed without considering the data on its ceRNA. In particular, we found that the plasma levels of miR‐331 decreased in lymph node‐positive BC and increased with recurrence.

The significant correlation between the plasma levels of H19 and miR‐675 that we detected in our investigations is due to the fact that miR‐675 is derived from and processed out of H19 (Cai and Cullen, 2007). In our study, the levels of H19 were increased in the plasma of BC patients, in particular its increase could be observed in the lymph node‐negative and aggressive subtype of HER2‐positive BC compared with healthy women. In contrast to its precursor lncRNA H19, we detected no deregulated levels of miR‐675 in the entire BC cohort as well as in the single BC cohorts. Our findings suggest that the excision of miR‐675 out of H19 occurs more infrequently in our BC population. However, we observed that the levels of miR‐675 significantly differed between nodal‐positive and nodal‐negative status, and along with the levels of H19, they differed between HER2‐positive subtype and the other subtypes. Interestingly, in HER2‐positive patients, H19 and miR‐675 concentrations were significantly higher and lower, respectively, indicating, again, a relationship between H19 and miR‐675. So far as we know, there are numerous publications on H19 measurements, whereas only one study exists on assessment of the expression status of miR‐675 in BC patients. In this study, Zhai *et al*. showed that the levels of miR‐675 were significantly upregulated in formalin‐fixed paraffin‐embedded (FFPE) tissue from BC patients compared with controls, but they did not differ between lymph node‐positive and node‐negative BC. These inconsistent data indicate that the miR‐675 levels in plasma do not reflect its expression in FFPE tissues. In line with our data, there are some studies showing increased plasma levels of H19 in BC patients (Jiao *et al*., 2018; Yu *et al*., 2018; Zhang *et al*., 2016), and similar to our study, Yu *et al*. detected significant differences in H19 concentrations between TNBC and non‐TNBC in a bivariate meta‐analysis model (Yu *et al*., 2018). In addition, our cell culture experiments show that H19 stimulated proliferation and inhibited apoptosis in MCF‐7 cells, whereas miR‐675 had no effect on both processes. However, miR‐675 mimic could alleviate the inhibitory effect on cell proliferation by siRNA H19. Discrepant data were obtained for the apoptotic effect of miR‐675 on H19. Curiously, both overexpression and inhibition of miR‐675 alleviated the apoptotic effect by siRNA H19.1 – although the experiment was repeated twice and an opposite effect was expected. Possibly, an interaction between cotransfected H19 and miR‐675 could explain this conflict in data. Analog to our findings Berteaux *et al*. also demonstrated the oncogenic character of H19. They reported that H19 promoted cell proliferation through the G1‐S transition, as the H19 promoter is activated by E2F1 in BC cells (Berteaux *et al*., 2005), while Li *et al*. found that H19 promoted proliferation and invasion of BC cells through the miR‐152/DNA methyltransferase 1 axis (Li *et al*., 2017a). Furthermore, Sun *et al*. (2015) described H19 as an estrogen‐inducible gene that plays a key role in survival and estrogen‐induced proliferation of MCF‐7 cells. Si *et al*. (2016) detected that H19 attenuated cell apoptosis in response to paclitaxel treatment by inhibiting the transcription of pro‐apoptotic genes BIK and NOXA. As reported by Vennin *et al*. (2015), the aggressive phenotype of BC cells may depend on the increased cell proliferation and migration *in vitro*, as well as the increased tumor growth and metastasis *in vivo* by the overexpression of H19/miR‐675.

Whereas there is a positive relationship between the levels of H19 and miR‐675 triggered by processing of miR‐675 out of its precursor H19 (Cai and Cullen, 2007; Tsang *et al*., 2010), there is a physical interaction and a reciprocal repression feedback loop between NEAT1 and miR‐204 (Lu *et al*., 2016). In our study, we detected that the plasma levels of NEAT1 were upregulated in the entire BC patient cohort. Notably, the levels of NEAT1 were upregulated in lymph node‐positive and TNBC patients. Along with miR‐204, the levels of NEAT1 differed between HER2‐positive status and the other respective status. A significant upregulation of NEAT1 was observed in tumor tissues of BC patients by several previous studies (Arshi *et al*., 2018; Choudhry *et al*., 2015; Li *et al*., 2017b; Zhang *et al*., 2017b; Zhao *et al*., 2017). Similar to our findings, Zhang *et al*. (2017b) observed that high expression of NEAT1 in BC tissues was closely related to lymph node metastasis and stimulated cell proliferation. Likewise, Zhao *et al*. detected that NEAT1 expression was significantly upregulated in BC tissues compared to adjacent normal tissues and that higher NEAT1 expression was positively associated with lymph node metastasis. They also reported that NEAT1 promoted cell invasion and proliferation by negatively regulating miR‐218 in BC (Zhao *et al*., 2017). Moreover, Choudhry *et al*. (2015) demonstrated that induction of NEAT1 in hypoxia led to accelerated cellular proliferation, improved clonogenic survival, and reduced apoptosis. As well, Ke *et al*. (2016) showed that downregulation of the expression of NEAT1 induced apoptosis in BC cells. These and our findings indicate that NEAT1 seems to be an oncogene. On the other hand, Shen *et al*. (2017) revealed that miR‐204 acts as a tumor suppressor, since miR‐204 suppressed the malignant behavior of BC cells. Wang *et al*. (2015) reported that overexpression of miR‐204 inhibited proliferation and promoted apoptosis in BC cells by targeting the Janus kinase 2 (JAK2). In addition, Li *et al*. (2014) discovered a decreased miR‐204 expression in tissues of BC patients that was significantly associated with tumor stage, metastasis, and survival. Our BC patient cohort displayed no deregulated levels of miR‐204 in plasma, but cotransfected miR‐204 could alleviate the inhibitory effect on cell proliferation by siRNA NEAT1. The effect on apoptosis by downregulated NEAT1 was weaker than that by overexpressed miR‐204. However, the effect on apoptosis by cotransfected siRNA NEAT1 and miR‐204 mimic was similar to the effect by miR‐204 alone. Supplementing camptothecin to MCF‐7 cells supported further our findings, suggesting that NEAT1 affects cell proliferation and apoptosis in context of miR‐204, and pointing to a reciprocal repression feedback loop in BC as observed in nasopharyngeal carcinoma (Lu *et al*., 2016).

Finally, we also planned to investigate the relationship between HOTAIR and miR‐331. However, we obtained false‐positive data, possibly because of the too low plasma levels of HOTAIR. Our amplification curves showed that in most cases, the PCR products contained primer dimers products. Whereas its ceRNA HOTAIR was not detectable in plasma of our BC cohort, the deregulated amounts of miR‐331 that particularly correlated with lymph node status and recurrence could be detected in BC plasma. To date, the inverse correlation of the expression levels between HOTAIR and miR‐331, and the involvement of miR‐331 in the post‐transcriptional regulatory network of HOTAIR have been reported for cervical and gastric cancer (Liu *et al*., 2014; Zhang *et al*., 2017a; Zhou *et al*., 2014). Chang *et al*. showed that in hepatocellular carcinoma (HCC), a highly invasive tumor with a high recurrence rate, miR‐331 is one of the most significantly overexpressed miRNAs and highly associated with metastasis. In xenograft mice, the inhibition of miR‐331 resulted in marked inhibition of proliferation and metastasis of HCC (Chang *et al*., 2014). These and our findings demonstrate the involvement of miR‐331 in advanced and recurrent tumors.

## 5 Conclusion

In conclusion, our quantifications of plasma H19/miR‐675 and NEAT1/miR‐204 suggest a crosstalk between these lncRNAs and miRNAs that might drive BC development and progression. This crosstalk between lncRNAs and miRNAs further complicates the mechanism of post‐transcriptional regulation of gene expression. Based on these preliminary data, further analyses in a larger BC cohort as well as functional analyses in cell lines from different BC subtypes are needed to reveal the mechanisms of post‐transcriptional regulation in more detail. Notably, future targeted therapies should consider that there is an interplay between lncRNAs and miRNAs, and that they do not act alone to affect mRNA expression.

## Conflict of interest

The authors declare no conflict of interest.

## Author contributions

HS made the conception and design. HS participated in the development of methodology. BS performed the experiments. VM and LOF contributed to the acquisition of samples and clinical data. HS analyzed the data. HS wrote the manuscript. VM, LOF, and KP reviewed and/or revised the manuscript. All authors read and approved the final manuscript.

## Supporting information


**Table S1.** Sequences of ncRNA assays used in the study.

**Table S2.** Measured data of miRNA and lncRNA expression levels normalized and evaluated by the Δ*C*_t_ method.
