# Supplementary material for: Interplay of lncRNA H19/miR‐675 and lncRNA NEAT1/miR‐204 in breast cancer
Source: Mol Oncol. 2019 Mar 14;13(5):1137–49. doi: 10.1002/1878-0261.12472 (PMC6487715; doi:10.1002/1878-0261.12472)
Supplement: Supplementary file 1 — Table S1. Sequences of ncRNA assays used in the study. [file MOL2-13-1137-s001.docx]

**Supplementary Table 1.** Sequences of ncRNA assays used in the study

| **ncRNAs** | **Sequences** | **Companies** |
| --- | --- | --- |
| hsa-miR-484 | UCAGGCUCAGUCCCCUCCCGAU | Thermo Fisher |
| hsa-miR-204 | UUCCCUUUGUCAUCCUAUGCCU | Thermo Fisher |
| hsa-miR-331-3p | GCCCCUGGGCCUAUCCUAGAA | Thermo Fisher |
| hsa miR-675 | UGGUGCGGAGAGGGCCCACAGUG | Thermo Fisher |
| Syn-cel-miR-39-3p | UCACCGGGUGUAAAUCAGCUUG | Qiagen |
| Syn-hsa-miR-204-5p mimic | UUCCCUUUGUCAUCCUAUGCCU | Qiagen |
| Syn-hsa-miR-675-5p mimic | UGGUGCGGAGAGGGCCCACAGUG | Qiagen |
| Anti-hsa-miR-675-5p Inhibitor | UGGUGCGGAGAGGGCCCACAGUG | Qiagen |
| Hs-H19.1 siRNA | ACCGCAATTCATTTAGTAGCA | Qiagen |
| Hs-H19.2 siRNA | CAGCCTTCAAGCATTCCATTA | Qiagen |
| Hs-H19.3 siRNA | CTGCACTACCTGACTCAGGAA | Qiagen |
| Hs-H19.4 siRNA | CTCCACGGAGTCGGCACATA | Qiagen |
| Hs-NEAT1.1 siRNA | GCCGGGAGGGCTAATCTTCAA | Qiagen |
| Hs-NEAT1.2 siRNA | TTGCCTATCTAGTATCTTCAA | Qiagen |
| Hs-NEAT1.3 siRNA | AACTTGAACTTTACTTCGTTA | Qiagen |
| Hs-NEAT1.4 (TncRNA_3) siRNA | CTGCGTCTATTGAATTGGTAA | Qiagen |
