# Supplementary material for: Interplay of lncRNA H19/miR‐675 and lncRNA NEAT1/miR‐204 in breast cancer
Source: Mol Oncol. 2019 Mar 14;13(5):1137–49. doi: 10.1002/1878-0261.12472 (PMC6487715; doi:10.1002/1878-0261.12472)
Supplement: Supplementary file 2 — Table S2. Measured data of miRNA and lncRNA expression levels normalized and evaluated by the ΔC t method. [file MOL2-13-1137-s002.docx]

**Supplementary Table 2.** Measured data of miRNA and lncRNA expression levels normalized and evaluated by the ΔCt method

| No | **2^(ΔCt) H19 Reference ß-Actin** | **2^(ΔCt) miR-675 Reference cel-miR-39 +miR 484** | **2^(ΔCt) HOTAIR Reference ß-Actin** | **2^(ΔCt) miR-331 Reference cel-miR-39 +miR-484** | **2^(ΔCt) NEAT1 Reference ß-Actin** | **2^(ΔCt) miR-204 Referenz celmiR-39 +miR 484** |
| --- | --- | --- | --- | --- | --- | --- |
| Breast cancer patients | | | | | | |
| 1 | 0.228986323 | 0.041330005 | not measurable | 0.02579675 | not measurable | 0.001039424 |
| 2 | 0.053412948 | 0.004062734 | 0.010003625 | 0.03089106 | 0.009662865 | 0.000412496 |
| 3 | 0.069348092 | 0.009322935 | not measured | 0.00721392 | 0.03103414 | 0.000561536 |
| 4 | 0.03287571 | 0.000472194 | not measured | 0.01966354 | 0.02201234 | 0.000286668 |
| 5 | 0.069508505 | 0.005492454 | not measured | 0.0031328 | 0.03563514 | 0.003874789 |
| 6 | 0.007758535 | 0.004249965 | not measured | 0.00228277 | 0.002535818 | 0.000198992 |
| 7 | 0.040479445 | 0.000541782 | not measured | 0.00307898 | 0.006143742 | 0.000134199 |
| 8 | 0.071297732 | 0.001429771 | not measured | 0.00390625 | not measurable | 0.002280132 |
| 9 | 0.037507771 | 0.018262145 | 0.004955799 | 0.00794906 | 0.006539216 | 0.004086269 |
| 10 | 0.07898616 | 0.024348893 | not measurable | 0.00984313 | 0.004227855 | 0.003456034 |
| 11 | 0.059265377 | 0.004602606 | not measurable | 0.00950784 | 0.03189434 | 0.004314273 |
| 12 | 0.045227164 | 0.002823434 | 0.000247549 | 0.01158445 | 0.03103414 | 0.003536815 |
| 13 | 0.058449453 | 0.003043612 | not measurable | 0.00486504 | 0.000102412 | 0.011868927 |
| 14 | not measurable | 0.00191293 | not measurable | 0.02777633 | 0.000116289 | not measurable |
| 15 | 0.041330005 | 0.000616619 | not measurable | 0.01002676 | 0.000733288 | not measurable |
| 16 | 0.03883048 | 0.002162127 | 0.022718321 | 0.00754638 | 0.001056372 | 0.004865038 |
| 17 | 0.087171479 | 0.002977533 | not measurable | 0.00576552 | 0.003262062 | 0.003412394 |
| 18 | 0.152830035 | 0.006920059 | not measurable | 0.00446126 | not measurable | 0.001141384 |
| 19 | 0.070969027 | 0.003781917 | not measurable | 0.0112807 | 0.003843581 | 0.001797242 |
| 20 | 0.058992145 | 0.001584599 | not measurable | 0.00565993 | 0.006115417 | 0.001754165 |
| 21 | 0.050531728 | 0.004196299 | not measurable | 0.01360235 | 0.000399828 | 0.003239529 |
| 22 | 0.022250784 | 4.76664E-05 | not measurable | 0.08537752 | 0.000709948 | 0.001939634 |
| 23 | not measurable | 0.00298787 | 0.055041617 | 0.03081977 | not measurable | not measurable |
| 24 | 0.251738888 | 0.00467225 | not measurable | 0.00168271 | 0.000959786 | 0.00183933 |
| 25 | 0.028955877 | 0.000239393 | not measurable | 0.05225333 | 0.000683392 | not measurable |
| 26 | 0.021893807 | 8.09094E-05 | not measurable | 0.01495391 | 0.000181635 | 0.003050652 |
| 27 | 0.013952492 | 0.000217003 | not measurable | 0.06683125 | not measurable | 0.000886249 |
| 28 | 0.027016788 | 0.000501429 | not measurable | 0.00336541 | 0.001708169 | 7.38522E-05 |
| 29 | 0.1900878 | 0.000502009 | not measurable | 0.00101803 | not measurable | 1.32529E-05 |
| 30 | 0.111619939 | 0.001272311 | not measurable | 0.002791 | 0.00319493 | 0.000751295 |
| 31 | 0.09635969 | 0.002248741 | not measurable | 0.0024213 | not measurable | 0.00068735 |
| 32 | 0.041044517 | 0.001401966 | not measurable | 0.0065771 | 0.004006801 | 0.000839412 |
| 33 | 0.012090352 | 2.79202E-05 | 0.002031367 | 0.01090903 | 0.005348438 | 1.40409E-05 |
| 34 | 0.012750226 | 0.001149323 | not measurable | 0.00116537 | 0.017217267 | 0.000813633 |
| 35 | 0.068235517 | 0.004058043 | not measurable | 0.00687226 | 0.006059159 | 0.000989052 |
| 36 | 0.116091426 | 0.000642804 | not measurable | 0.00045035 | 0.022982295 | 0.00093484 |
| 37 | 0.040386026 | 0.002489376 | 0.006215129 | 0.00728932 | 0.004898877 | not measurable |
| 38 | 0.029977004 | 0.001384263 | not measurable | 0.01705888 | 0.002036066 | 0.001756192 |
| 39 | 0.031177881 | 0.002886093 | not measurable | 0.00581233 | 0.004732003 | 0.002117631 |
| 40 | 0.067140791 | 0.001466575 | 0.000969817 | 0.00207405 | 0.002382458 | 7.04357E-05 |
| 41 | 0.015770075 | 0.000353337 | 0.001487048 | 0.01350839 | 0.007976657 | 0.000917503 |
| 42 | 0.031980434 | 0.003299966 | not measurable | 0.01112539 | 0.00538925 | 0.001018033 |
| 43 | 0.036651092 | 0.003452044 | not measurable | 0.00445096 | 0.002382458 | 0.000365375 |
| 44 | 0.014311645 | 0.000504917 | not measurable | 0.01792769 | 0.009865902 | 0.000835542 |
| 45 | 0.073641696 | 0.006976246 | 0.110848924 | 0.00737402 | 0.00109869 | 0.006769821 |
| 46 | 0.018928012 | 0.002325355 | not measurable | 0.00448192 | 0.000819293 | 0.000238289 |
| 47 | 0.022405551 | 0.001253346 | not measurable | 0.00847052 | 0.000708309 | 0.003247023 |
| 48 | 0.009464006 | 0.023573885 | not measurable | 0.0221482 | 0.004044004 | 0.00273041 |
| 49 | 0.02056973 | 0.001062491 | not measurable | 0.05691686 | 0.009957505 | 0.004389686 |
| 50 | 0.018928012 | 0.002233208 | not measurable | 0.00125915 | 0.006129563 | not measurable |
| 51 | 0.08153871 | 0.001077323 | not measurable | 0.01780385 | 0.00255843 | 0.001240382 |
| 52 | 0.040666933 | 0.001722039 | not measured | 0.01230169 | 0.007425308 | 0.000717367 |
| 53 | 0.013508394 | 0.001243251 | not measured | 0.00433927 | 0.010946904 | 0.000691332 |
| 54 | 0.022148201 | 0.002355093 | not measured | 0.00689612 | 0.003416339 | 0.000947666 |
| 55 | 0.01713789 | 0.000898621 | not measured | 0.00825795 | not measurable | 0.000573998 |
| 56 | 0.01753847 | 0.00061876 | not measured | 0.02636924 | 0.002344236 | 0.001698331 |
| 57 | 0.067140791 | 0.001008668 | not measured | 0.01841042 | not measurable | 0.001219073 |
| 58 | 0.0074597 | 0.00109996 | not measured | 0.01890616 | 0.003480073 | 0.000409173 |
| 59 | 0.01790699 | 0.002379708 | not measured | 0.00660756 | not measurable | 0.001363629 |
| 60 | 0.02490899 | 0.000648023 | not measured | 0.0145282 | 0.00092231 | 0.001302049 |
| 61 | 0.003150944 | not measurable | not measured | 0.11357102 | 0.005785537 | 0.005886667 |
| 62 | not measurable | 6.76691E-07 | not measured | 0.01528577 | 0.00356849 | 0.000360345 |
| 63 | 0.051000905 | 4.61492E-05 | not measured | 0.00598956 | 0.007425308 | 0.000692131 |
| Healthy women | | | | | | |
| 1 | 0.03032532 | 0.000962006 | not measured | 0.04475936 | 0.00909469 | 0.001845635 |
| 2 | 0.03388209 | 0.000866007 | not measured | 0.03265241 | 0.00531733 | 0.003586188 |
| 3 | 0.011517728 | 0.003839143 | not measured | 0.01384012 | not measurable | 0.000337771 |
| 4 | 0.025737219 | 0.003075422 | not measured | 0.01661158 | not measurable | 0.001523565 |
| 5 | 0.014544996 | 0.005435644 | not measured | 0.01286861 | 0.000419221 | 0.001164021 |
| 6 | 0.03699139 | 0.002529965 | not measured | 0.01117692 | not measurable | not measurable |
| 7 | 0.020617311 | 0.009131072 | not measured | 0.01830439 | 0.000906964 | 0.000801505 |
| 8 | 0.064405751 | 0.003721242 | not measured | 0.01841042 | 0.001426472 | 0.001387465 |
| 9 | 0.027776334 | 6.93858E-05 | not measured | 0.00828662 | 0.000639841 | 0.001159994 |
| 10 | 0.03883048 | 0.005268714 | not measured | 0.03589682 | not measurable | not measurable |
